# Heart rate changes after phlebotomy in polycythaemia vera and healthy donors: An observational case‐crossover pilot study

**DOI:** 10.1111/tme.70038

**Published:** 2025-10-23

**Authors:** Rik P. B. Tonino, Elisabeth M. J. Huis in 't Veld, Martin R. Schipperus, Jaap Jan Zwaginga

**Affiliations:** ^1^ Haematology Department Leids Universitair Medisch Centrum Leiden The Netherlands; ^2^ TRIP, Transfusion and Transplantation Reactions in Patients Leiden The Netherlands; ^3^ Internal Medicine Department Groene Hart Ziekenhuis Gouda The Netherlands; ^4^ Department of Donor Medicine Research Sanquin Amsterdam The Netherlands; ^5^ Department of Medical Affairs Sanquin Amsterdam The Netherlands

**Keywords:** biosensors, blood donation, haemoglobin, heart rate, phlebotomy

## Abstract

**Background:**

Haemoglobin plays a crucial role in oxygen transport, and any acute deviation will trigger compensatory hemodynamic functions. While the consequences of anaemia are well documented, the effects of haemoglobin reduction in individuals without anaemia remain less explored. Patients with polycythaemia vera and healthy blood donors, who both undergo regular phlebotomies, offer a valuable model for studying these effects.

**Methods:**

This observational case‐crossover study assessed short‐term physiological and quality‐of‐life changes following phlebotomy in five patients with polycythaemia vera and six healthy blood donors. Participants were remotely monitored using a smartwatch and completed daily quality‐of‐life assessments. The primary outcome was heart rate, while secondary outcomes included step count and quality‐of‐life measures.

**Results:**

Patients with polycythaemia vera exhibited stable heart rates, with only minor variations in physical activity and quality of life after phlebotomy. In contrast, healthy blood donors experienced a significant increase in heart rate, which returned to baseline within a week. Physical activity remained clinically unchanged in both groups, and quality‐of‐life scores were stable.

**Conclusions:**

This pilot study demonstrates that any acute haemoglobin reduction, even within the normal range, induces measurable heart rate changes that are directly related to probably the most optimal oxygen delivery state. Moreover, our studies show that wearable technology is sensitive enough to detect these effects. Hence, this nowadays readily available telemetry allows monitoring of subtle physiological changes in a research setting, but it also offers a path towards optimising QoL for patients with anaemia, polyglobulia and for blood donors.

## 1 BACKGROUND

Low haemoglobin levels can lead to impaired oxygen delivery, and consecutively to fatigue, dyspnoea, lack of concentration and therewith a reduced quality of life.^1^, ^2^, ^3^ Even in healthy blood donors, frequent donations cause an increase in symptoms like tiredness, dyspnoea, and restless legs.^4^ To combat these adverse outcomes, RBC transfusions are used to improve functional outcomes in anaemic patients.^5^, ^6^, ^7^ However, excessively high haemoglobin levels, such as in polycythaemia vera (PV), can cause hyperviscosity, potentially limiting oxygen transport.^8^ Normal levels are maintained between 12.1 and 15.1 g/dL in females and 13.8 and 17.2 g/dL in males.

The relationship between haemoglobin mass and physiological effects, especially cardiorespiratory outcomes, remains insufficiently explored in non‐anaemic populations.^9^ In athletic contexts, strategies such as altitude training and the use of erythropoiesis‐stimulating agents have been employed to enhance Hb mass and thereby increase maximal oxygen uptake.^10^, ^11^, ^12^ Nonetheless, the optimal Hb concentration for maximising physiological function remains unclear. Similarly, the appropriate target Hb level in anaemic patients has long been a subject of clinical debate. Investigating the relationship between Hb mass and physiological effects across different clinical settings and baseline Hb levels may provide valuable insights into the optimisation of haemoglobin.

Blood donors and patients with PV, who undergo regular therapeutic phlebotomies, present a valuable model for studying these effects. The regulated removal of 500 mL of blood allows for precise assessment of its impact on various physiological parameters without further interventions. In healthy donors, an average reduction in Hb of 8.8% ± 1.9% has been observed post‐donation.^13^ Indeed, previous studies of healthy donors show changes in heart rate and VO_2_max 24–48 h post‐phlebotomy,^9^, ^14^ whereas no comprehensive evaluations of physiological outcomes in PV patients undergoing phlebotomies have been documented.^15^

Given these observations, the aim of this exploratory study is twofold: firstly, to investigate the impact of haemoglobin mass reduction on heart rate, physical function, and quality of life in patients with PV as well as healthy blood donors, and secondly to explore whether current wearable techniques are sensitive enough to detect these effects.

## 2 METHODS

### 2.1 Study design and recruitment

This observational case‐crossover pilot study assessed the physiological and quality‐of‐life changes associated with phlebotomy in patients with PV and healthy donors in the Netherlands.

Patients with PV were identified by their treating physicians at the Leiden University Medical Centre and referred to the local investigator (author R.T.). Donors were recruited from Sanquin, the national not‐for‐profit organisation responsible for the collection and distribution of blood and plasma. Donors who were invited to participate in another study (FAINT^16^—a parallel study conducted by author E.H.i.tV., investigating predictors of vasovagal reactions to needles) were invited to also participate in the current study.

Eligibility criteria for both groups included adults aged 18 years or older. For PV patients, exclusion criteria included poor functional status (ECOG ≥3), the presence of arrhythmias or significant cardiac conduction disorders, a life expectancy of less than 3 months, and concurrent participation in other clinical trials likely to interfere with this study. Patients with secondary polycythaemia or patients who were scheduled for oncological treatments or surgeries during the study period were also excluded. This study adhered to the principles of the Declaration of Helsinki and complied with the General Data Protection Regulation.

### 2.2 Data collection and procedures

Following enrolment, participants were provided with a smartwatch by the local site investigator. They were instructed to wear the device continuously, beginning 1 week prior to the intervention and continuing until 1 week following the intervention. The seven‐day period preceding each intervention was designated as the baseline monitoring phase. Continuous data collection resumed immediately post‐intervention and continued for an additional 7 days, constituting the follow‐up period.

Data collection for PV patients was conducted over three phlebotomy sessions per participant. However, for the healthy donors, data collection was limited to a single blood donation session due to logistical constraints, as the FAINT study did not follow donors over repeated donations. All phlebotomies were conducted as part of standard care, with no alterations to clinical procedures. The questionnaires were sent on a daily basis to the private e‐mail addresses of the participants through CASTOR EDC, an electronic data management platform.

#### 2.2.1 Heart rate measurements

Continuous heart rate and step count monitoring was conducted using the Withings Steel HR smartwatch, a commercially available wearable device previously employed by the authors in both completed and ongoing clinical trials.^5^ Biosensor data were transmitted through Withings' GDPR‐compliant platform. Continuous data were processed to obtain daily median heart rates and total daily step counts. In addition, mean heart rates were calculated separately for nighttime and daytime periods to account for potential confounding effects of physical activity on daytime heart rate measurements.

#### 2.2.2 Quality of life

Participants completed a daily quality‐of‐life assessment using the EQ‐5D‐5L questionnaire, which evaluates five dimensions: mobility, self‐care, usual activities, pain/discomfort, and anxiety/depression. Each dimension is rated on a 5‐point scale, ranging from no problems to extreme problems, producing a 5‐digit health state profile. These responses were converted into an EQ‐5D‐5L index score, adjusted for the Dutch population (range: −0.329 to 1.000). In addition, participants rated their overall health using the EQ visual analogue scale (VAS; range: 0%–100%), a self‐reported measure ranging from ‘the best health you can imagine’ to ‘the worst health you can imagine’. To adjust for possible activity‐related confounding in heart rate outcomes, we also asked our participants to complete an activity VAS (0%–100%) ranging from ‘the least active you can imagine’ to ‘the most active you can imagine’.

#### 2.2.3 Haemoglobin levels

Haemoglobin levels were measured prior to each intervention as part of routine clinical assessments for both PV patients and healthy donors via venipuncture and fingerpick measurement, respectively.

#### 2.2.4 Demographic information and confounders

Additional demographic data, *β*‐blocker usage, and adverse reactions were collected for confounding and baseline characterisation.

### 2.3 Statistical analysis

The sample size calculation indicated a requirement of nine participants per group; however, due to logistical constraints of this low‐budget study, this pilot study included 5 PV patients and 6 healthy donors.

To minimise variability, each participant acted as their own control due to the case‐crossover design; repeated measures across three phlebotomy sessions further strengthened the analysis. To mitigate potential bias from natural variability, as well as the effects of a particular day that may not be reflective of daily life (e.g., a visit to a theme park), heart rate and activity data were summarised per 3 days for analysis. The day of phlebotomy was excluded from analysis, as the procedure induces acute volume depletion and puncture‐related stress, both of which are known to transiently affect heart rate through mechanisms unrelated to the physiological effects under investigation.

Primary and secondary endpoints were analysed using linear mixed models with random intercepts to account for repeated measures. Statistical significance was set at *p* < 0.05.

A linear mixed‐effects model with random intercepts for participants was used to assess the effect of phlebotomy on median heart rate, with fixed effects for time (baseline vs. 1–3 days and 4–7 days post‐phlebotomy), sex, and *β*‐blocker usage. Days −1 to −3 were used as baseline; days <−3 were used as a control period.

## 3 RESULTS

A total of 55 patients scheduled for phlebotomy were screened, of whom 42 were excluded due to indications other than PV, four did no longer need phlebotomies, two patients only incidentally required phlebotomies, and two patients declined participation. Of 444 healthy donors invited, 25 participated in the FAINT study; six of these also enrolled in this add‐on study. Ultimately, 11 participants were enrolled, comprising 5 PV patients who were stable on regular phlebotomy (at least every 4 months) and six healthy donors. Inclusion started July 19th, 2022, and follow‐up ended March 20th, 2023. Baseline data are presented in Table 1.

**TABLE 1:** Baseline characteristics.

	Polycythaemia vera	Healthy donors
	*n* = 5	*n* = 6
Age, median (range)	64 (48–72)	50 (34–56)
Female gender, *n* (%)	1 (20%)	6 (100%)
B‐blocker use, *n* (%)	2 (40%)	1 (17%)
Phlebotomies, cumulative	13	6
Pre‐phlebotomy haemoglobin level, median (range)	15.1 g/dL (13.2–17.9)	14.0 g/dL (13.1–14.8)

### 3.1 Data collection

Following excellent compliance, sufficient day‐ and nighttime data were collected throughout the observation period (day −7 to 7). On day −7, data acquisition was incomplete, with two daytime and one nighttime intervals lacking sufficient measurements due to delayed device initiation, preventing calculation of a valid mean heart rate. No further data loss or connectivity issues occurred.

### 3.2 Primary outcome: Heart rate

**FIGURE 1:**
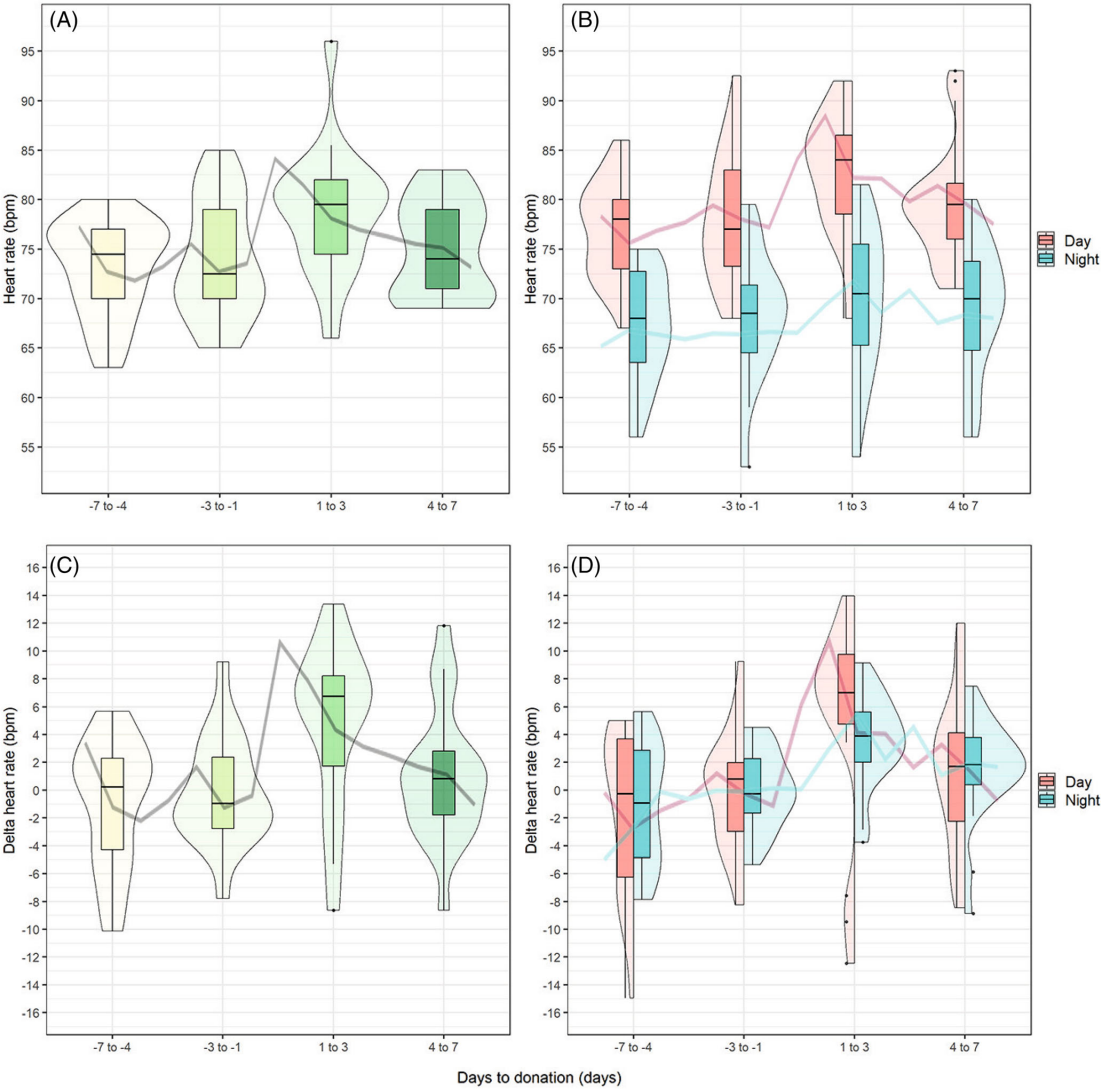
Donor heart rates: Violin and boxplots summarising the median heart rates per 3 days (A) and stratified for day (06:00–23:59) and night (00:00–05:59) (B) and Δheart rates (C and D). The line graphs correspond to the median heart rates per day. Day 0 was not included in the violin and boxplots due to the direct phlebotomy‐related confounding effects on heart rate.

**FIGURE 2:**
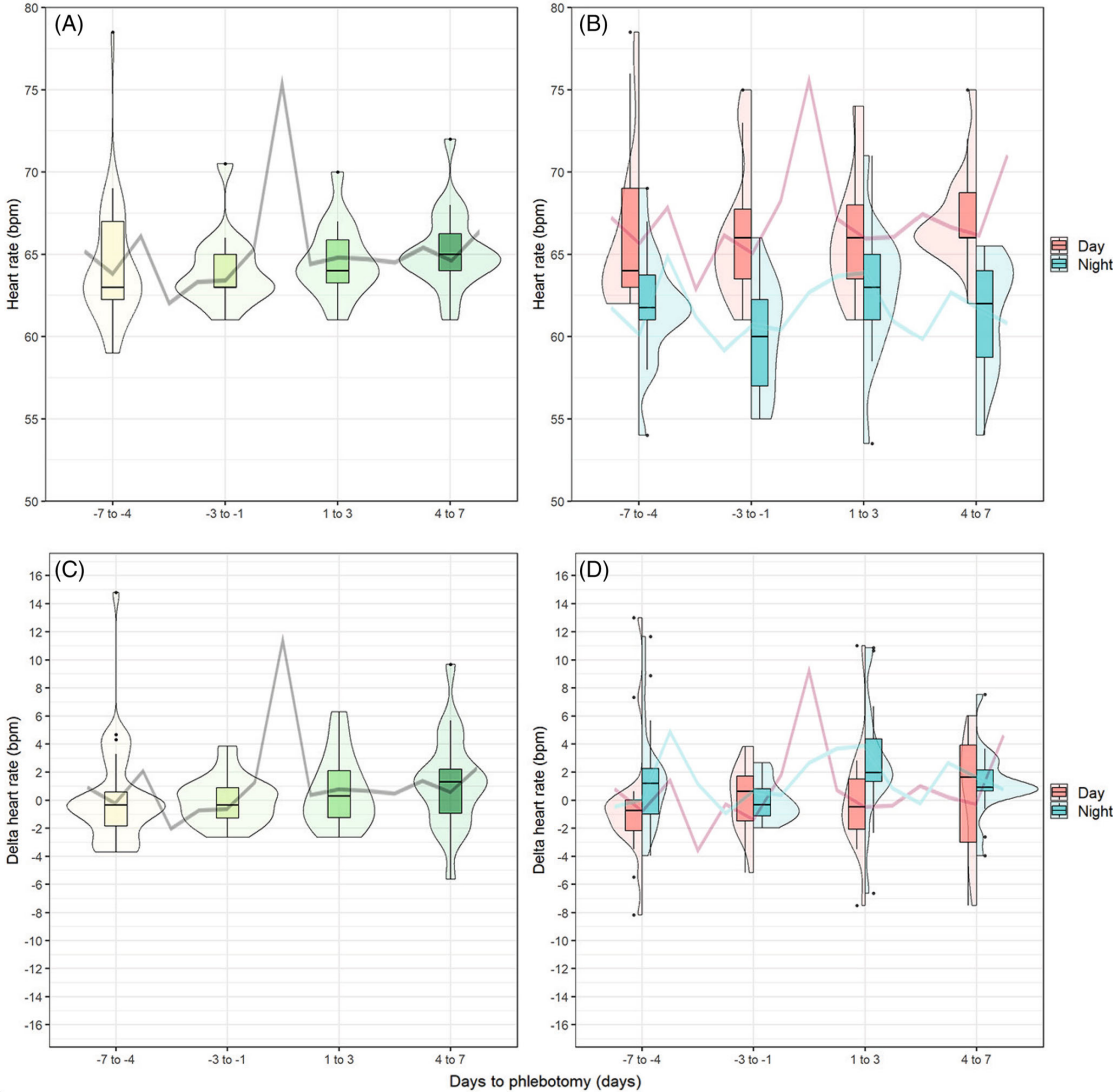
Phlebotomy heart rates: Violin‐ and boxplots summarising the median heart rates per 3 days (A) and stratified for day (06:00–23:59) and night (00:00–05:59) (B) and Δheart rates (C and D). The line graphs correspond to the median heart rates per day. Day 0 was not included in the violin‐ and boxplots due to the direct phlebotomy‐related confounding effects on heart rate.

As illustrated in Figure 1, healthy donors exhibited a significant increase in median heart rate during the immediate post‐donation period (days 1–3), with an estimated change of *β* = 4.9 bpm (95% CI: 2.4 to 7.5; *p* < 0.001), followed by a return to near‐baseline levels by days 4–7. Absolute heart rate values are presented in Table 2, and the results of the linear mixed model analysis are detailed in Table 3. This pattern was observed consistently in both daytime and nighttime heart rate measurements (*p* = 0.008 and *p* = 0.010, respectively), with a more pronounced increase noted during daytime hours. In contrast, heart rate in patients with polycythaemia vera remained stable throughout the observation period, ranging from 63 to 65 bpm, with only minor variation between daytime and nighttime measurements (Figure 2).

**TABLE 2:** Outcomes.

			Control (D‐7–D‐4)	Baseline (D‐3–D‐1)	Visit 1 (D1–D3)	Visit 2 (D4–D7)
Donor	Heart rate (bpm, median)	Total	75 (70–77)	73 (70–79)	80 (75–82)	74 (71–79)
		Day	78 (73–80)	77 (73–83)	85 (78–88)	80 (76–82)
		Night	67 (61–73)	68 (62–71)	71 (65–76)	70 (64–74)
	Steps (amount/24 h)		6650 (2433–8327)	5394 (3521–8999)	6679 (4890–9450)	5612 (2991–9189)
	QoL (range: −0.33 to 1.00)	EQ‐5D‐5L index score	1.00 (0.87–1.00)	1.00 (0.87–1.00)	1.00 (0.87–1.00)	1.00 (1.00–1.00)
		VAS‐QoL	0.9 (0.82–0.94)	0.92 (0.87–1.00)	0.84 (0.73–0.93)	0.91 (0.78–1.00)
		VAS‐Activity	0.64 (0.59–0.8)	0.60 (0.51–0.70)	0.71 (0.61–0.82)	0.70 (0.61–0.84)
Polycythaemia vera	Heart rate (bpm, median)	Total	63 (62–67)	63 (63–65)	64 (63–66)	65 (64–67)
		Day	64 (63–69)	66 (63–69)	66 (63–69)	67 (66–69)
		Night	62 (60–64)	60 (56–63)	63 (61–65)	62 (58–64)
	Steps (amount/24 h)		4990 (3172–6569)	5319 (3800–6159)	3992 (2656–5396)	4472 (2897–5926)
	QoL (range: −0.33 to 1.00)	EQ‐5D‐5L index score	0.89 (0.88–1.00)	0.92 (0.88–1.00)	0.91 (0.88–1.00)	0.89 (0.85–1.00)
		VAS‐QoL	0.70 (0.67–0.80)	0.72 (0.70–0.77)	0.72 (0.68–0.80)	0.71 (0.70–0.82)
		VAS‐Activity	0.70 (0.61–0.71)	0.70 (0.65–0.75)	0.70 (0.62–0.80)	0.74 (0.70–0.79)

**TABLE 3:** Linear mixed model outcomes for heart rate.

Donors					
Parameter	*β*	SE	95% CI	*p*‐value	Estimated marginal means
Intercept	73.89	0.91	72.1–75.7	<0.001	
Control	−0.86	1.28	−3.4–1.7	0.502	73.0
Baseline	*ref*	*ref*	*ref*	*ref*	73.9
Day 1–3	4.94	1.29	2.4–7.5	<0.001	78.8
Day 4–7	1.18	1.22	−1.2–3.6	0.337	75.1

Polycythaemia vera					
Parameter	*β*	SE	95% CI	*p*‐values	Estimated marginal means
Intercept	63.63	0.563	62.5–64.7	0.000	
Control	0.23	0.655	−1.1–1.5	0.725	64.1
Baseline	*ref*	*ref*	*ref*	*ref*	64.3
Day 1–3	0.63	0.692	−0.7–2	0.362	64.7
Day 4–7	1.23	0.647	−0.1–2.5	0.061	65.3
*β*‐blocker	0.5	0.511	−0.5–1.5	0.326	
Sex	0.35	0.624	−0.9–1.6	0.573	

*Note*: Baseline = days −3 to −1; control = days −7 to −4; Day 0 was excluded due to direct phlebotomy effects on heart rate like volume depletion and puncture induced stress; In the donor group, all participants were female and none were using *β*‐blockers. Therefore, sex and *β*‐blocker use were not included in the model.

### 3.3 Secondary outcomes: Physical activity and quality of life

**FIGURE 3:**
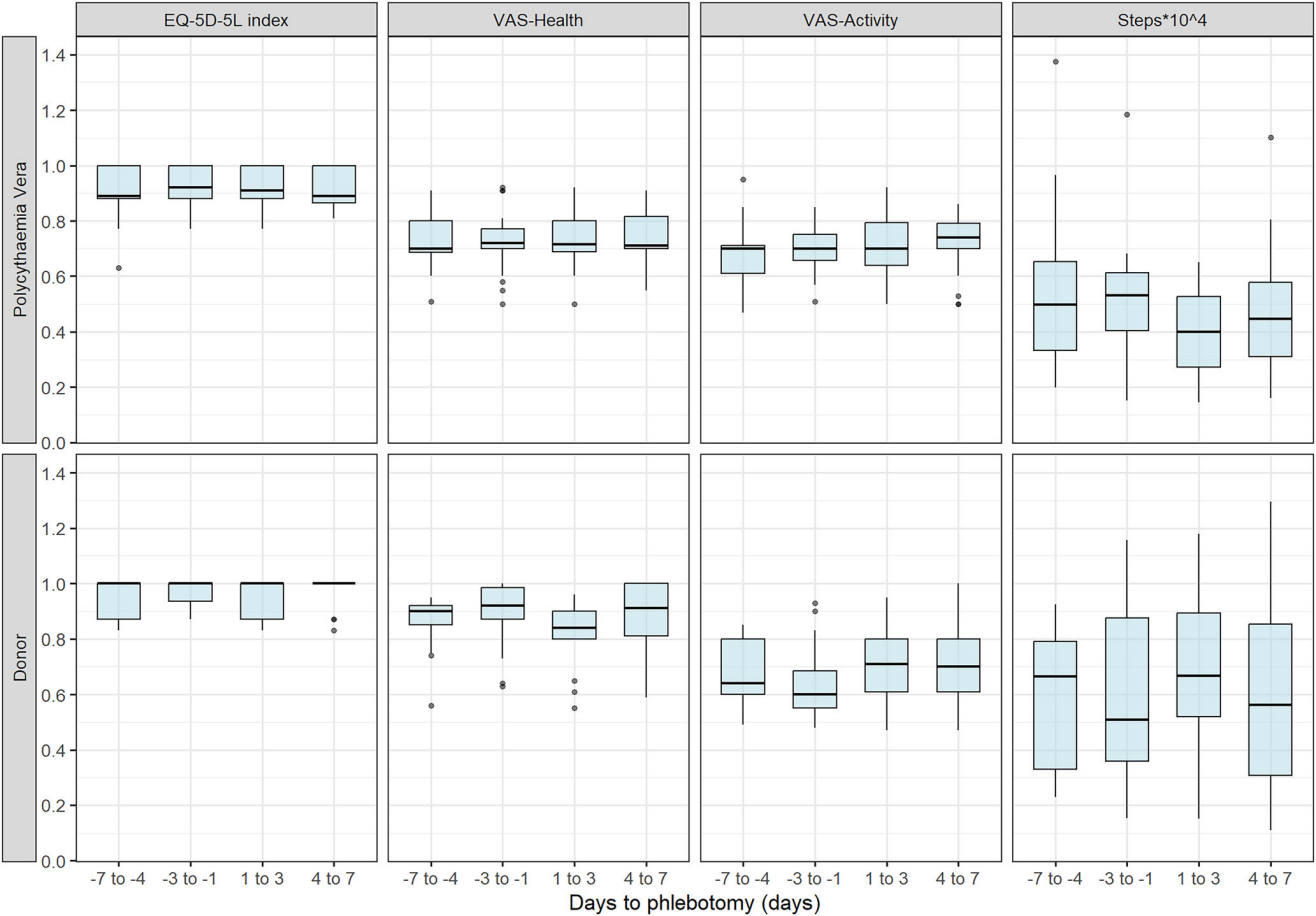
Boxplots summarising secondary outcomes per 3 days.

As depicted in Figure 3, step counts slightly, non‐significantly, increased from baseline to day 1–3 before declining slightly to baseline at day 4–7 in healthy donors. PV patients showed an opposite trend, with step counts decreasing post‐phlebotomy, followed by a partial recovery at day 4–7.

EQ‐5D‐5L index scores remained stable in healthy donors (1.00 across all periods), whereas PV patients had lower baseline QoL scores (0.70, IQR: 0.65–0.75) with slight improvement post‐phlebotomy. VAS‐based QoL ratings in donors declined from 0.92 to 0.84 post‐phlebotomy but returned at day 4 to 7 (0.91). PV patients maintained stable VAS‐QOL scores (0.89–0.92) across all periods. VAS‐activity ratings in donors increased post‐phlebotomy, whereas they remained stable in PV patients.

## 4 DISCUSSION

This study aimed to explore the short‐term physiological and quality‐of‐life effects of phlebotomy in PV patients and healthy donors. However, due to resource constraints, recruitment was prematurely halted, resulting in a limited sample size of 11 participants. While this restricts the generalizability of findings, the data provide valuable insights into the short‐term physiological effects of haemoglobin reduction. Notably, the outcomes demonstrated that the wearable technology used was sensitive enough to detect even small haemoglobin mass reductions within the normal range, highlighting its potential for larger‐scale future research and validation of changes in more patient‐specific treatments.

Our primary outcome, heart rate, demonstrated a transient physiological response to phlebotomy in both PV patients and healthy donors. However, a key distinction was observed between the two groups: while PV patients exhibited no sustained heart rate elevation post‐phlebotomy, healthy donors experienced a prolonged increase lasting almost up to 1 week.

While the PV and donor groups cannot be compared due to the small sample size and baseline differences, the observed difference in heart rate response to phlebotomy is intriguing. We therefore present the following as an exploratory consideration: this difference in heart rate response might be explained by their respective baseline haemoglobin levels (Table 1). Healthy donors—who had lower haemoglobin levels at baseline than PV patients—experienced a more pronounced and sustained increase in heart rate after phlebotomy. Another possible explanation is that donors experienced higher stress levels than PV patients seems less likely because the sustained increase in heart rate for nearly a week in blood donors is atypical for such a transient stress response.

The transient heart rate elevation observed on the day of phlebotomy in both groups is likely attributable to the acute volume depletion rather than haemoglobin reduction, as PV patients' heart rates returned to baseline within 24 h—the plasma volume is typically completely replenished within 24–48 h.^17^ The sustained elevation in healthy donors interestingly suggests that even modest reductions in haemoglobin within the normal range can lead to measurable hemodynamic changes, especially so because this sustained elevation was not found in the PV group, potentially due to reduced oxygen‐carrying capacity. This aligns with the timing of restoration of the Hb mass; the duration of complete restoration of Hb‐mass has been documented to be between 20 and 59 days, although restoration starts in the first week.^13^

Phlebotomy in PV is primarily prophylactic, aimed at preventing hyperviscosity‐related complications rather than alleviating symptoms. While it has been suggested that reducing hyperviscosity may lower cardiac workload and heart rate, this effect was not observed in our treated cohort, likely due to their minimal baseline symptom burden as they were stably treated. Hypothetically, in newly diagnosed PV patients—with higher baseline Hb masses—this effect might be measurable.

Consistent with this, quality‐of‐life assessments showed no clinically significant differences between groups or periods. A slight improvement in EQ‐5D scores observed in PV patients during Period 4 may suggest a delayed symptomatic benefit of phlebotomy. However, given the small sample size, this finding should be interpreted with caution.

Similarly, step count measurements fluctuated across study periods, but the variability was substantial, making it unlikely that the observed differences reflect clinically meaningful changes in physical activity levels. These findings suggest that while phlebotomy effectively manages hematologic parameters in PV, its immediate impact on quality of life and physical activity remains limited.

### 4.1 Study limitations and future directions

The primary limitation of this study is the small sample size, which restricts the ability to draw definitive conclusions. Furthermore, the lack of diversity, with 6/6 donors being female, restricts generalizability. Additionally, the absence of comparable studies makes it challenging to contextualise the findings. Furthermore, an adequate comparison between donors and PV patients would require comparable baseline characteristics. To better detect phlebotomy‐induced correction of suboptimal oxygen delivery, PV patients with even more supraphysiological Hb and Ht values—for example, those starting therapy and not in maintenance like our cohort—should be considered. Likewise, to investigate QoL and physical exercise changes as a function of suboptimal oxygen transport and the correction of the latter, we do not necessarily need many more patients but certainly longer prospective observation periods starting with the initiation of phlebotomy treatment. For the observed and reported changes in HR, however, patients being their own control, it is not likely that higher sample sizes would alter our conclusions. Additionally, Hb measurements at predefined moments, like day 3 and 7, could be helpful to correlate future studies.

Despite these limitations, our findings show that even minor haemoglobin reductions can elicit measurable physiological responses in healthy individuals, while stable and chronically treated PV patients may be less affected due to their higher baseline haemoglobin levels. These findings contribute to our understanding of haemoglobin mass changes across diverse clinical contexts. Notably, wearable technology proved sensitive enough to detect these effects. Hence, this nowadays readily available telemetry not only allows monitoring subtle physiological changes in research settings but it also offers a path towards optimising QoL for patients with anaemia, polyglobulia and for blood donors.

## AUTHOR CONTRIBUTIONS

R.T., M.S., and J.J.Z. conceived the study design. R.T., J.J.Z., and E.H.V. participated in the inclusion of patients. The study was carried out by R.T. R.T., M.S., and J.Z. participated in the interpretation of the results. R.T. wrote the first draft. All authors reviewed the draft.

## CONFLICT OF INTEREST STATEMENT

The authors have no competing interests.

## PATIENT CONSENT STATEMENT

Informed consent from all participants was obtained.

## Data Availability

The data that support the findings of this study are available from the corresponding author, R.T., upon reasonable request.
